# Physical therapy and opioid use for musculoskeletal pain management: competitors or companions?

**DOI:** 10.1097/PR9.0000000000000827

**Published:** 2020-09-24

**Authors:** Steven Z. George, Adam P. Goode

**Affiliations:** Department of Orthopaedic Surgery, Duke Clinical Research Institute Duke University, Durham, NC, United States

**Keywords:** Musculoskeletal pain, Nonpharmacological management, Physical therapy, Opioid use

## Abstract

Musculoskeletal (MSK) pain conditions are highly prevalent and a leading cause of disability globally. When people with MSK pain seek health care, they often receive treatment not aligned with best practices, including initial management options such as opioids. In recent practice guidelines, nonpharmacological treatments have been emphasized for initial pain management, and physical therapists are providers who routinely deliver nonpharmacological treatments. The purpose of this review is to describe the current and future state for how physical therapy may be used to increase exposure to nonpharmacological treatments for MSK pain conditions. For the current state, we review existing observational evidence investigating early exposure to physical therapy and its influence on subsequent opioid use. For the future state, we propose clinical research questions that could define the role of physical therapy on interdisciplinary teams working towards improving effectiveness of nonpharmacological treatments through more rigorous study designs. These clinical questions are intended to guide health services research and clinical trials when building an evidence base of nonpharmacological care options for MSK pain conditions.

## Abstract 2

Existing evidence is reviewed and gaps in the literature are identified to better understand how physical therapy has been used to provide exposure to nonpharmacological treatments.

## 1. Introduction

Musculoskeletal (MSK) pain is highly prevalent, representing the largest subset of chronic pain conditions,^26^ and is a leading cause of disability globally.^21^ People seeking health care can be offered initial treatment options not aligned with pain management best practices.^36^ Receiving an opioid prescription for initial treatment of MSK pain is one example of a practice not aligned with current practice guidelines.^13,37^ In the United States, opioids accounted for 18.8% of medication prescriptions for chronic low back pain and 76.9% of the opioid prescriptions were for long-term use.^38^ People with newly diagnosed MSK pain who were opioid-naive when seeking care and subsequently received an opioid prescription had increased risk of chronic opioid use (defined as ≥10 prescriptions or ≥120 days supply between 91 and 365 days after the initial diagnosis).^35^ The risk of transitioning from opioid-naive to chronic opioid use for newly diagnosed MSK pain was highest for people with low back pain or with multiple areas of pain (eg, knee and low back pain).^35^ Reliance on opioid medication for MSK pain management has led to calls for a shift to providing care models that support nonpharmacologic therapies.^22^

As part of their routine scope of practice, physical therapists deliver nonpharmacological treatments when caring for people with MSK pain conditions. Many of the nonpharmacological treatments (eg, exercise, body-based manual therapies, transcutaneous electric nerve stimulation, and physical agents) used by physical therapists are supported by current practice guidelines.^37^ There is variation in how these treatments are used by physical therapists, but generally they are first delivered with a goal to modulate pain, and then with a goal to facilitate functional gains that allows return to daily activities as well as vocational and social roles. Use of physical therapists to deliver nonpharmacological treatments is supported by practice guidelines, but there are data available from the United States suggesting it may be underutilized for MSK pain conditions. In the National Ambulatory Medical Care Survey, opioid prescriptions for new chronic MSK pain conditions were present in 21.5% of visits, whereas prescriptions for physical therapy were only 10.0% of visits.^14^ Therefore, there is potential for physical therapists to play an increased role in the delivery of nonpharmacological treatment for people with MSK pain.

The purpose of this review is to describe the current and future state for how physical therapy may be used to increase exposure to nonpharmacological treatments for MSK pain conditions. For the current state, we review existing observational evidence investigating early exposure to physical therapy and its influence on subsequent opioid use. For the future state, we propose clinical research questions that could define the role of physical therapy on interdisciplinary teams working towards improving pain management outcomes by incorporating nonpharmacological treatments as an alternative and/or adjunct to opioid use. These clinical questions are intended to add to existing observational studies by building an evidence base consisting of more rigorous study designs (eg, randomized and/or pragmatic clinical trials) that investigate the effectiveness of nonpharmacological care options for MSK pain conditions.

## 2. Musculoskeletal pain and opioids: impact and response for the United States

The U.S. healthcare system is facing numerous challenges with healthcare inefficiencies. In particular, inadequate care for MSK pain conditions can have tremendous societal consequences leading to decreased quality of life and increased costs. It is the primary reason for 1 in 8 persons reporting lost workdays due to MSK pain conditions and direct costs equate to 5.2% of the U.S. gross domestic product—$796.3 billion dollars—annually.^40^ Furthermore, the number of people with MSK pain conditions is increasing, placing higher demands on the healthcare system.^40^ In response to the concerns regarding MSK pain and opioid usage, several high-profile public and private organizations in the United States have developed guidelines for the treatment of noncancer pain. These include the Federal Drug Administration Education Blueprint for Health Care Providers Involved in the Management or Support of Patients with Pain (2017),^16^ The Joint Commission on Accreditation of Health Care Organizations new and revised pain assessment and management standards for hospitals (2017),^28^ and the American College of Physicians Clinical Practice Guideline on Low Back Pain (2017).^37^ All these organizations have found the evidence sufficient enough to support use of multiple nonpharmacological therapies as preferred options for patients with noncancer pain. However, uptake of these clinical guidelines is slow^3^ and prescriptions for opioids for MSK pain remains common.^4,17^

Clinical guidelines recommend nonpharmacological treatments as frontline care for MSK conditions but referrals to physical therapy for evaluation and management are disproportionate to the number in need.^23^ For example, in the United States, referrals to physical therapy from primary care range between 7% and 20%^15,18^ for low back pain and 13.6% for knee osteoarthritis.^2^ For some common MSK conditions, such as low back pain and knee osteoarthritis, encounters within the healthcare system have greatly increased; however, referrals to physical therapy have remained stable or have decreased, whereas opioid prescriptions have increased.^2,27,34^ There are likely complex reasons for the low rate of physical therapy referrals, including geographic, cultural/linguistic, and financial barriers to access, in addition to differences in the opinions and perspectives of primary care providers on the value of PT care. The variation in the number of allowable PT visits by insurance providers and, in some cases, the high out-of-pocket copays or coinsurance may also deter patients from seeking care from a physical therapist or limit provider referrals to physical therapy.^6^ Opportunities to improve care of MSK pain conditions by improving access to nonpharmacological providers, decreasing barriers to access, and implementing innovative delivery models can potentially reduce pain, improve function, and decrease opioid exposure.

## 3. Exposure to physical therapy and association with opioid use

**Figure 1.**
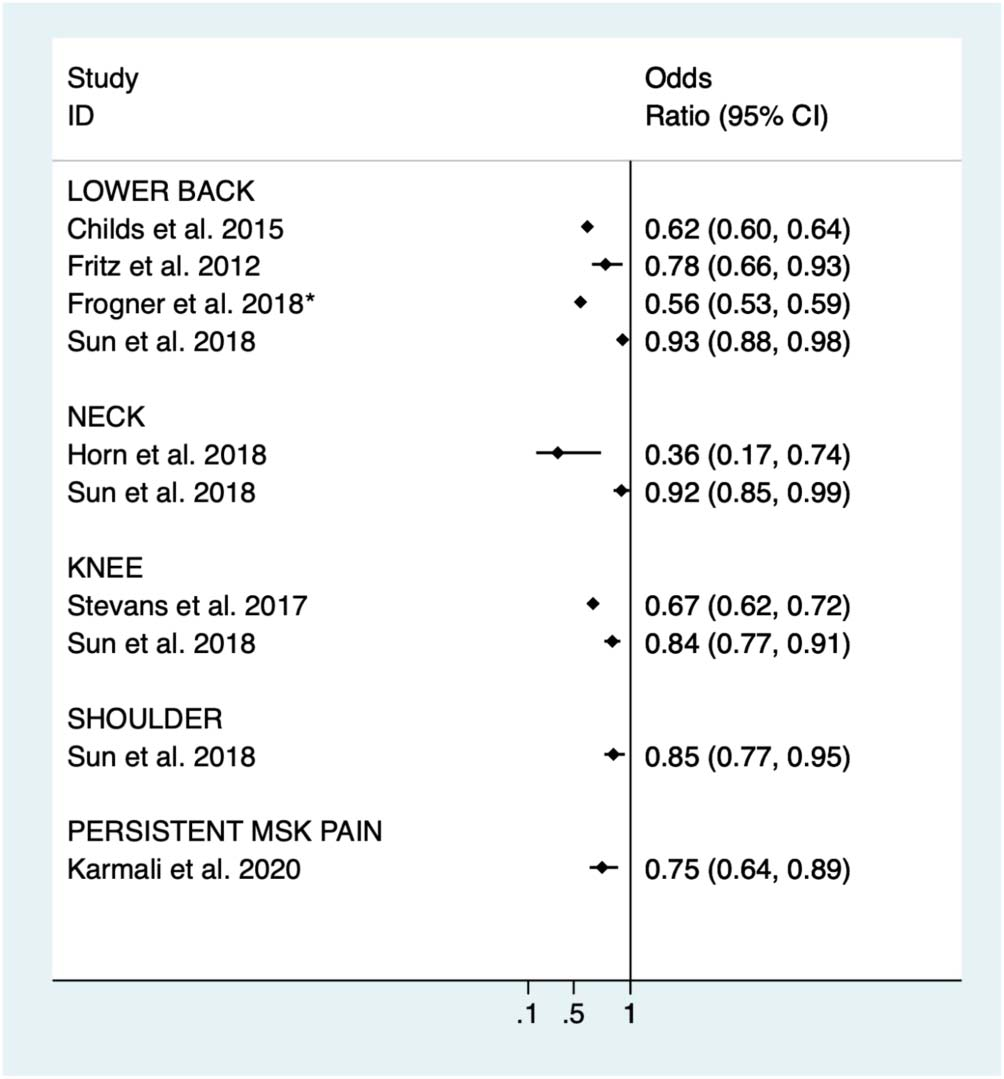
Illustration of the early exposure to physical therapy and subsequent opioid prescriptions among observational studies. All are adjusted estimates unless specified. *Frogner et al. conducted a general linear model approach. Raw counts were abstracted and used for the unadjusted odds ratio. The adjusted and instrumental variable approach results are reported in the text of this article. MSK, musculoskeletal.

Several observational studies have examined whether early access to physical therapy influences opioid prescriptions. In the United States, “early access” to physical therapy services has been defined in many different ways, and it is beyond the purpose of this review to go into detail on any specific definition.^33^ Typically early access relates to moving the first appointment with physical therapy closer to when a patient first accesses the healthcare system for pain management. Observational studies of MSK pain conditions^8,41^ as well as a published systematic review on low back pain^1^ highlight recent studies that have examined early exposure to physical therapy with subsequent opioid prescriptions. We abstracted adjusted data reported from these studies and created a visualization for reporting odds ratios and 95% confidence intervals for these estimates (using Stata v16, College Station, TX). Some studies varied in their analytical techniques and the variables included for confounding adjustment. Therefore, we highlighted these differences in the figure caption. To avoid confusion with this being perceived as a meta-analysis that is typically accompanied with a systematic review of the literature, we reported individual estimates without a summary estimate across studies (Fig. 1). Other reasons supporting reporting of only individual estimates included the between-study variation in methodological and analytical approaches, different patient populations, and multiple outcomes definitions used. Figure 1 clearly illustrates the consistency (ie, same side of the null value) of effects that is found across these studies.

Most studies examining the relationship between early physical therapy and opioid exposure have included participants with low back pain.^8,18,19,41^ However, recently this work has expanded to include those with neck,^24,41^ knee,^39,41^ shoulder pain,^41^ and persistent MSK pain.^29^ One study included in Figure 1 implemented advanced analytical techniques to address common biases found in observational designs. Frogner et al.^19^ reported an 89.4% decreased probability of an opioid prescription if they had seen a physical therapist first for low back pain rather than late or never; their approach used an instrumental variable relating to distance to a provider.

There is considerable convergence that early exposure to physical therapy reduces the odds of future opioid prescriptions. This convergence occurs despite differences in MSK populations studied, including opioid-tolerant and opioid-naive participants, the inherent challenges associated with observational designs, wide variability in analytical approaches, and using different definitions of early vs late physical therapy. In the United States, these observational studies have spurred health systems to reconsider the placement of physical therapy for patients receiving nonpharmacological care for MSK pain conditions.^20^ However, there are also notable gaps and weaknesses of this literature base with the most notable being that the evidence to date is exclusively from observational designs involving secondary analyses of claims or registry data. Although some of these studies have taken steps to decrease potential biases with advanced analytical approaches, observational designs have limited ability to account for biases that could inflate the protective influence of physical therapy on opioid prescription.^33^ Studies that take a pragmatic trial approach, as opposed to sole reliance on observational designs, with randomization at the clinic level, may provide an effective alternative to controlling bias and provide higher-quality evidence on the impact of improving access to physical therapy for nonpharmacological interventions for MSK pain conditions.^20^

## 4. Innovative musculoskeletal care delivery models

There are several novel care models that move physical therapy to the forefront of the patient experience. Many of these novel healthcare delivery models being piloted for MSK pain conditions have been previously implemented within other medical conditions. In the United States, the high prevalence of depression among patients seeking primary care led to new models of colocated and integrated primary care.^11^ Colocation, for example, has proven successful for mental-health services, where it has almost doubled the rate of guideline based care,^32^ facilitating collaboration due to proximity of specialized services.^44^ When comparing physical therapists who have been colocated within primary care practice to standard referral from primary care to offsite physical therapy, there has been a 12% reduction in opioid prescribing and 9.6% reduction in emergency department visits.^7^ In addition, integration of providers within a single practice has been a proposed model for improving the access and delivery of services. Ongoing work by Carvalho et al.^5^ is examining the effects of integrating physical therapy within a primary care practice with physical therapists providing frontline evaluation and treatment for patients with a chief complaint of MSK pain. In this scenario, the entire clinic is organized so that patients entering the practice are first seen by a physical therapist for nonpharmacological approaches and then referred to the primary care physician for any needed pharmacologic interventions. This integrated approach may be a better way to bring nonpharmacological treatment to the forefront of the patient experience, either alone or in combination with pharmacological options.

Patients are taking more control over decisions about their care in today's healthcare environment. An interesting care delivery model from Denninger et al.^12^ examined the effects of patient choice of their first provider for an MSK complaint. Patients in a privately insured group health plan were provided access to 1 of 8 clinics in a 3-U.S. county region where they had the choice to have care from a physical therapist or a medical provider. Patients choosing to access physical therapy first for their MSK pain condition had lower total healthcare costs, when compared to those who had selected to start with usual medical care.^12^ The effects on opioid prescriptions were not reported in this initial description of the care delivery model, but future work in patient choice models may help to identify a specific type of patient who is more likely to be engaged and accepting of nonpharmacological treatment for MSK pain conditions.

As more third-party payers recognize the value in early exposure to physical therapy services, some third-party payers are providing patients with the option to seek care without a referral from a medical provider and reimburse for treatment. The evidence for these changes comes from the recent observational studies examining the association between exposure to nonpharmacological provider first and the effect on opioid prescriptions. Kazis et al.^30^ and Horn et al.^25^ reported large reductions in opioid prescriptions when patients would see a nonpharmacological provider first. The nonpharmacological providers included in these analyses were chiropractors, acupuncturists, and physical therapists and in some cases the analysis highlighted differences amongst these providers for influencing healthcare utilization beyond opioid use. For example, Horn et al.^25^ reported that frontline care by chiropractors, but not physical therapists, reduced advanced spine imaging, radiography, and injections downstream up to 12 months after the first visit. Collectively, these innovative care models suggest that early referral to a nonpharmacological provider continues to be a strong indicator for decreasing opioid prescriptions for MSK pain conditions, and some variation in other healthcare utilization (eg, imaging studies) is expected depending on the type of nonpharmacological provider seen.

The overarching goal for these models is to improve all aspects of healthcare quality—efficiency, effectiveness, equity, patient-centeredness, safety, and timeliness. In the midst of the opioid crisis, the concerns with safety are often a higher priority, and the ability of these models to limit opioid exposure while still providing effective, patient-centered options for pain management will determine the long-term sustainability of these approaches.

## 5. Clinical research questions

There is potential for earlier access to physical therapy and the implementation of innovative care delivery models to improve delivery of nonpharmacological care. Increased implementation of nonpharmacological options is likely to be driven by clinical practice guideline recommendations^37^ and patient preference for providers, such as physical therapists, who deliver nonpharmacological treatments.^12^ The goal of improving nonpharmacological delivery is to not to totally eradicate opioid use, but rather to provide comprehensive pain management for patients with MSK pain conditions, which includes pharmacological and nonpharmacological options. Important clinical research questions remain regarding the effectiveness of physical therapy when the goal is to provide nonpharmacological treatments as part of a broader pain management strategy. Three such unanswered research questions are described below.

### 5.1. Physical therapy as an adjunct to other nonpharmacological or pharmacological approaches

One clinical research question relates to determining whether physical therapy provides better pain relief when used in conjunction with other nonpharmacological or with pharmacological (ie, opioids or other nonnarcotic pain-relieving medications) treatments. This question relates to how physical therapy is often not prescribed alone and is used in combination with other approaches. One example of investigating multiple nonpharmacologic approaches (ie, physical therapy and mental health) for persistent MSK pain conditions comes from an analysis of U.S. Medicare beneficiaries with no opioid prescriptions in the past 6 months. In this analysis, early use of mental health services was associated with decreased odds of low-risk opioid use (adjusted odds ratio [aOR] = 0.81; 95% confidence interval [CI] 0.68–0.96), but higher odds of long-term opioid use (95% aOR = 1.28–2.90).^29^ By contrast, early use of physical therapy was associated with decreased odds of long-term opioid use (aOR = 0.75; 95% CI 0.64–0.89) but greater odds of high daily opioid dose (aOR = 1.15; 95% CI 1.15–1.36).^29^ These data highlight the potential complexities in comparing different approaches. Beyond retrospective analyses that focus on outcomes of opioid usage, such as the aforementioned example, very little is known about the efficacy or effectiveness of multimodal approaches for pain reduction and functional improvements.^36^ Typically, clinical trials are designed to compare unimodal treatment, and are more likely to provide efficacy and effectiveness data on pharmacological to pharmacological comparisons or nonpharmacological to nonpharmacological comparisons. Therefore, there are very few properly controlled trials in this area, and the evidence base is limited.^36^

(1) Unanswered clinical question—does physical therapy provide additional pain relief when used in combination with other nonpharmacological or opioid and nonopioid pharmacological approaches?

### 5.2. Physical therapy for opioid taper or cessation

Another clinical research question relates to determining whether physical therapy is a useful adjunct when the treatment goal is opioid taper or cessation. In this situation, the physical therapist would be part of an interdisciplinary team, with a primary role of delivering nonpharmacological pain management options. In many ways, this team would function in a similar manner as already mentioned when innovative care models were reviewed; however, this team has a much more specific goal of reducing opioid use. There is variability in which other disciplines are part of this team, but one framework reported in the literature described a core team that included the patient and healthcare providers from physical therapy, behavioral health, primary care, complementary services, and selected specialists.^43^ The physical therapist on this team must be aligned with the overall team goals, given the complexity of decreasing or stopping opioid usage, especially for patients with signs that are consistent with opioid use disorder. The aforementioned framework included multiple pillars to provide the structure necessary to develop a unified plan of care and facilitate communication across the interdisciplinary team. These pillars include *public health*, *clinical knowledge*, *neuroscience*, *education*, *wellness*, *mental health*, and *patient characteristics*.^43^ The complexity of this clinical question would have to be taken into consideration when designing research to test the effectiveness of a given care pathway. That is, there would have to be control in the type of providers included on the team, clear definitions for what each provider role was during the study period, best practices in accounting for people who do not complete the pathways, and what are the other outcomes of interest besides decreasing opioid usage. This complexity likely contributes to why there is such a small evidence base in this area because these studies are difficult to design, implement, and successfully complete. However, it will be necessary to generate data that are rigorous enough that it can be used to inform future clinical pathways in this area.

(1) Unanswered clinical question—does physical therapy improve the success rate of interdisciplinary care pathways designed to taper or cease opioid usage?

### 5.3. Unintended consequences of increased exposure to physical therapy

The third clinical research question has to do with unintended consequences of increasing exposure to physical therapy. The aforementioned observational data indicate advantages of increasing exposure to physical therapy for reduction of opioid prescriptions for many common MSK pain conditions (Fig. 1). However, altering healthcare systems to increase exposure to physical therapy could create problems that would need to be balanced against the benefit of reducing opioid use.^33^ Earlier access to physical therapy may not prevent additional healthcare utilization in areas other than opioid prescriptions. This was reported in the study from Horn et al.^25^ where having a physical therapist as first provider for neck pain reduced opioid prescriptions over the next 12 months, but did not reduce injections, advanced imaging, or radiography. The impact of exposure to earlier physical therapy on healthcare utilization outcomes other than opioid prescriptions should be the focus of future observational studies and clinical trials because these data will provide a better understanding of how risk of healthcare utilization may (or may not) change. Furthermore, these future studies will be planned and completed in the context of already knowing that many of the individual nonpharmacological treatments being delivered by the physical therapists have equivocal evidence for their effectiveness.^9,10^ Existing and emerging evidence indicating better healthcare utilization outcomes should not be parlayed into providing supporting evidence for the effectiveness of physical therapy on other pain-related outcomes (eg, pain intensity or self-report of function). Finally, there is no evidence suggesting that physical therapists are any more or less guideline adherent for care delivery compared to other provider types. Therefore, increasing exposure to physical therapy could add unwarranted variability in care received for MSK pain conditions. Obviously, this additional variability would not include opioid prescriptions, but there are other ways physical therapy can deliver low value or ineffective care that would have to be considered when increasing exposure to physical therapy.^33^

(1) Unanswered clinical question—what are the unintended consequences for healthcare utilization, cost of care, and patient outcomes when increasing exposure to physical therapy for the primary goal of reducing opioid use?

## 6. Conclusion

This review described how physical therapy may be used to increase exposure to nonpharmacological treatments for people with MSK pain conditions. There are strong indications that future pain management will involve nonpharmacological treatment as a central component.^13,36,37^ These management models may require new care delivery models, an updated evidence base, and new payment policies that support use of nonpharmacological treatments.^20,22^ Progress can be made through use of more rigorous designs and collaboration among U.S. national agencies. For example, there is a large National Institutes of Health, Veteran's Administration, and Department of Defense funding initiative supporting pragmatic clinical trials investigating the effectiveness of nonpharmacological treatments for pain management.^31^ This review was largely supported by data from the U.S. system and as such, may contain challenges that are not relevant for other countries. However, a recent update from Traeger et al.^42^ indicated that similar challenges remain globally for health systems in delivering guideline-recommended physical and psychological therapies for low back pain. Ultimately, progress will be made through changes in health system delivery that facilitates provider behavior for delivering increased exposure to nonpharmacological treatment as part of a larger societal goal of providing adequate relief for those with MSK pain conditions.^36^

## Disclosures

The authors have no conflicts of interest to declare.

A.P. George and S.Z. Goode were supported by the National Institutes of Health/National Center for Complementary and Integrative Health while working on this manuscript (AT009790).
